# Modification by antioxidant supplementation of changes in human lung function associated with air pollutant exposure: A systematic review

**DOI:** 10.1186/1471-2458-11-532

**Published:** 2011-07-05

**Authors:** Amir Y Tashakkor, Katherine S Chow, Chris Carlsten

**Affiliations:** 1 The University of British Columbia, Vancouver, British Columbia, Canada

## Abstract

**Background:**

Outdoor air pollution, given its demonstrated negative effects on the respiratory system, is a growing public health concern worldwide, particularly in urban cities. Human exposure to pollutants such as ozone, nitrogen oxides, combustion-related particulate matter and oxides of sulfur is responsible for significant cardiopulmonary morbidity and mortality in both adults and children. Several antioxidants have shown an ability to partially attenuate the negative physiological and functional impacts of air pollutants. This study systematically presents current data on the potential benefits of antioxidant supplementation on lung function outcomes associated with air pollutant exposures in intact humans.

**Methods:**

Electronic databases (MEDLINE, EMBASE, BIOSIS Previews, Web of Sciences, Environmental Sciences & Pollution Management and TOXNET) were systematically searched for all studies published up to April 2009. Search terms relating to the concepts of respiratory tract diseases, respiratory function tests, air pollution, and antioxidants were used. Data was systematically abstracted from original articles that satisfied selection criteria for inclusion. For inclusion, the studies needed to have evaluated human subjects, given supplemental antioxidants, under conditions of known levels of air pollutants with measured lung function before and after antioxidant administration and/or air pollution exposure. Selected studies were summarized and conclusions presented.

**Results:**

Eight studies investigated the role of antioxidant supplementation on measured lung function outcomes after subject exposure to air pollutants under *controlled *conditions; 5 of these studies concluded that pollutant-induced airway hyper-responsiveness and diminution in lung function measurements were attenuated by antioxidant supplementation. The remaining five studies took place under *ambient *(uncontrolled) exposures and unanimously concluded that antioxidant supplementations attenuate the negative effects of urban air pollution on lung function.

**Conclusions:**

The data evaluating modification of changes in lung function associated with air pollutant exposure by antioxidant supplementation, in intact humans, is limited. Of 13 studies dedicated to this concern, ten demonstrated an attenuation of pollution-associated decrements. There is growing evidence for the benefit of anti-oxidant supplementation in moderating the effects of air pollution on lung function, but more research on human participants is needed to inform this topic.

## Background

Outdoor air pollution, particularly that produced by vehicle exhaust, has become a growing public health concern worldwide. Outdoor particulate matter (PM) air pollution is responsible for approximately 3% of adult cardiopulmonary disease mortality, 5% of respiratory cancer mortality, and 1% of mortality in children from acute respiratory infection in urban areas worldwide [1]. Air pollution can evoke symptoms in persons without underlying lung diseases and exacerbate symptoms in persons with lung diseases such as asthma and chronic obstructive pulmonary disease. For example, one study in 208 children showed that exposure to PM _2.5 _(PM with mean aerodynamic diameter of 2.5 microns or less) for an average of 22 weeks resulted in acute airway inflammation and decrease in Forced Expiratory Volume in 1 second, FEV_1 _(P = 0.048), and Forced Vital Capacity, FVC (p = 0.012) [2]. Similar studies have also demonstrated the negative effects of air pollution on lung function such as statistically significant decrements in FEV_1_, FVC, mid Forced Expiratory Flow (FEF_25-75_) and Peak Expiratory Flow (PEF) [3-5].

Data support the association between imbalanced oxidant homeostasis and lung disease including asthma [6]. Deficiencies in antioxidants have been associated with asthmatic severity and prevalence [7,8]. Furthermore, there is growing evidence for oxidative stress in response to air pollution [9]. Constituents in traffic-related air pollution, including quinones, aromatic hydrocarbons, and transition metals, have the potential to produce reactive oxygen species [10,11] and oxidative stress [12-15]; some of these effects may therefore, hypothetically, be reversible with antioxidants. A controlled human exposure model has shown increases in reduced glutathione levels (a sign of oxidative stress) in the bronchial and nasal airways following exposure to diesel exhaust [16]. Humans exposed to diesel exhaust have decreased glutathione and urate levels and depletion of glutathione is associated with the pathogenesis of idiopathic pulmonary fibrosis [17]. Animal studies have shown an increase in levels of antioxidant genes in epithelial cells after exposure to diesel exhaust particles [18].

However, there is a paucity of study regarding the ability of *supplemental *antioxidants such as Vitamin C and Vitamin E to prevent deterioration of lung function or other phenomena associated with air pollution. In addition, most literature on the current subject is based on animal or *in vitro *studies, while the quintessential proof of applicability necessitates human-based studies. Animal-modelled studies are limited in applicability by obvious physiological differences between the human and animal model, making them significantly limited in terms of public health recommendations. *In vitro *studies such as that performed by Greenwell et al. [19] are insightful but lack the direct evidence that is imperative for implementation into public health guidelines. Further, despite the informative nature of the broad literature and studies that investigate the effects of varying basal (endogenous) antioxidant concentrations on pollutant responsiveness [20], such studies are limited by concerns over confounding in observational studies (i.e., concerns that those with higher endogenous antioxidants are endowed with or prone to acquire beneficial factors other than the observed differences in antioxidants). Experimental studies, in which a single intervention is precisely administered, are particularly persuasive if an effect of the intervention is clearly observed.

Currently, there is particular interest on the role of supplemental antioxidants in ameliorating pollution-induced insults to the lung and a general recognition that there is insufficient evidence for a strong recommendation therein at this time [21]. The desire to best understand the relevant evidence for such a recommendation compelled this systematic review. Accordingly, we decided to focus on studies that included direct measurement of lung function, as changes in lung function are most commonly appreciated as clinically relevant whereas studies inclusive only of biomarkers may be insightful but less compelling in terms of recommendations to the public. Similarly, this systematic review did not evaluate studies that addressed the effects of endogenous antioxidants as modifiers of air pollution effects [22] or studies that addressed the effects of air pollution on altering endogenous antioxidant levels [23]; we have focused on the effects of exogenous administration of antioxidants, given the practical question of whether such supplements are likely to ameliorate air pollution-related respiratory health effects. In summary of the above, the current review addresses the evidence for the effects of antioxidant supplementation, in intact humans, on changes in lung function associated with measured levels of air pollution.

## Methods

The systematic search strictly focused on the effects of antioxidant supplementation in humans, both paediatric and adult, with respect to lung function as an outcome associated with quantified levels of air pollutants. Antioxidant supplementation could be naturally-occurring or synthetic, in any dose and form of administration, taken individually or in combination, and compared to placebo or to standard medication or care. Air pollutants were identified as ozone, nitrogen oxides, combustion-related particulate matter [excluding biomass and cigarette smoke], and oxides of sulfur. In summary, for inclusion the articles needed to have evaluated human subjects, given supplemental antioxidants, under conditions of known levels of air pollutants (amongst those listed above), with measured lung function before and after antioxidant administration and/or air pollution exposure.

**Figure 1:**
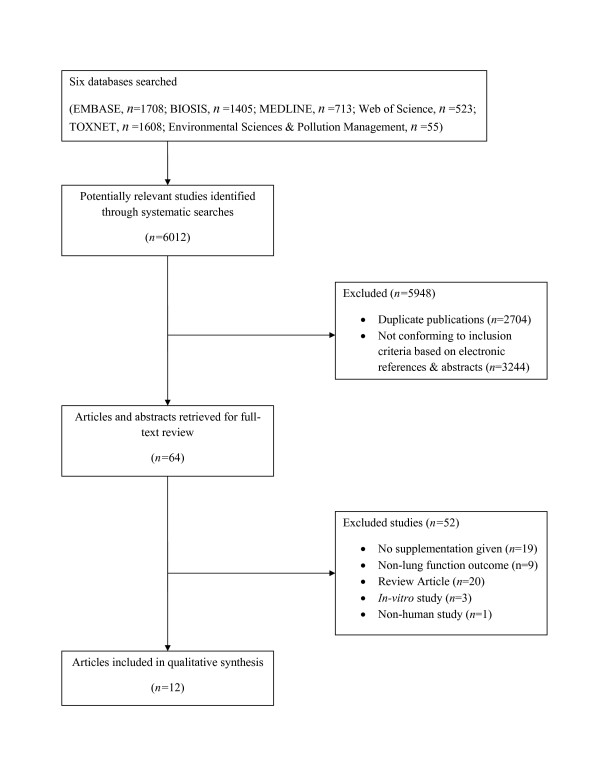
**Flow chart of included studies**.

As shown in Figure 1, since the research question encompasses topics in medicine, public health and environmental studies, the literature search included appropriate electronic databases. Peer-reviewed studies were identified from MEDLINE (1950 to April Week 4 2009), EMBASE (1980 to 2009 week 16), BIOSIS Previews (1969 to 2009), Web of Science (1965 to 2009 week 16), Environmental Sciences & Pollution Management (1967 to 2009 week 16), and TOXNET (1965 to 2009 week 16) databases. Search terms related to the concepts of respiratory tract diseases, respiratory function tests, air pollution, and antioxidants were used. They included MeSH (Medical Subject Headings) and keywords related to the concepts (Additional Files 1). Having developed the above literature search strategies, the searches were run on April 23, 2009.

Abstracts from this search were examined for duplicates, which were removed; the remaining abstracts were reviewed for inclusion based on title and abstract. There were no restrictions on participant age, study type or study language. Studies which investigated related phenomena, but not strictly lung function, such as exhaled nitric oxide [24] or medication use [25] were excluded. The first stage involved screening electronic abstracts. Authors CC and KC independently reviewed the abstracts, implementing predetermined selection criteria. Upon completion, both results were compared and abstracts which were included in one author's selection list and not the other were each discussed amongst the two authors. Upon review, abstracts were eliminated only after a unanimous decision, or (if disagreement remained) abstracts were kept for a full-text review. Full-text of the articles was obtained for the remaining abstracts and each was scrutinized applying the inclusion criteria. Further, references from selected studies were assessed for relevant articles, ensuring that the search was comprehensive.

After the full-text review, a total of thirteen studies consistent with our inclusion criteria were selected. One paper [26] described two independent studies, each of which met our specified criteria. Therefore, this paper was treated as two separate studies in the summarizing tables. Finally, selected studies were assigned to one of two tables, depending on whether participants were subject to controlled pollution as represented in Table 1 or ambient, uncontrolled (but measured) pollution conditions as represented in Table 2.

**Table 1:** Studies assessing effect of antioxidant supplementation on lung function associated with *controlled* air pollutant exposures in intact humans

First author, Year, Country	Study design	Conditions*	Pollutant exposure duration	Additional stresses during exposure	Population (N)	Age range	Inclusion / Exclusion criteria	Outcome
**Hackney, 1981, USA**	Randomized (non-crossover); supplement double-blinded; exposure nominally single-blinded	a) 800 IU Vitamin E (9 to 10 weeks); air then next-day ozone (0.5 ppm)	2 hr	Heat and "light exercise"	Healthy (34)	20-27	Not Specified.	No significant effect of antioxidants on ozone-attributable decrease in FEV_1 _and FVC. (i.e. statistical significance of supplementation effect: p > 0.05).
		b) Placebo (9 to 10 weeks);air then next day ozone (0.5 ppm)						
**Hackney, 1981, USA**	Randomized (non-crossover); supplement double-blinded, exposure nominally single-blinded	a) 1600 IU Vitamin E (11 to 12 weeks); air then ozone (0.5 ppm, for 2 subsequent days)	2 hr	Heat and "light exercise"	Healthy males (22)	22-28	Not Specified.	No significant effect of antioxidants on ozone-attributable decrease in FEV_1_, FVC, TLC, FEF_25%_, FEF_50% _(i.e. statistical significance of supplementation effect: p > 0.05).
		b) Placebo (11 to 12 weeks); air then ozone (0.5 ppm, for 2 subsequent days)						
**Chatham, 1987, USA**	Randomized (crossover); double-blinded	a) Vitamin C (1 gm, one hour before exposure) and Vitamin E (800 IU daily for unspecified duration); ozone (0.3 ppm)	2 hr	Exercise	Medical students (9)	21-29	Normal baseline pulmonary function, non-smokers, refrained from any drugs, including vitamin supplements.	Ozone-induced decrements in FVC were attenuated by vitamin C and vitamin E (p < 0.05), but no significant effect was observed in FEV_1_, FEF_25-75 _and SGaw (p > 0.05).
		b) Placebo; Ozone (0.3 ppm)						
**Mohensin, 1987, USA**	Randomized (crossover); double-blinded	a) Vitamin C (2000 mg/day for 3 days); NO_2 _(2.0 ppm)	1 hr		Healthy subjects (11)	18-36	Not Specified.	Airway hypersensitivity induced by NO_2 _was attenuated by pre-treatment with ascorbic acid, relative to placebo (p < 0.04).
		b) Placebo (3 days); NO_2 _(2.0 ppm)						
		c) Vitamin C (2000 mg/day for 3 days); air						
		d) Placebo; air						
**Peters, 2001,** **The Netherlands**	Double-blinded; cross over;	a) Placebo; ozone (0.4 ppm)	2 hr	Intermittent exercise	Mild atopic asthmatics (7)	19-26	Required a measurable plateau of the dose-response curve to methacholine, non smokers, no NSAID or vitamin use during study.	Airway hypersensitivity induced by O_3 _was attenuated by pre-treatment with apocynin, relative to placebo (p = 0.025).
		b) Apocynin (12 mg); ozone (0.4 ppm)						
**Samet, 2001, USA**	Randomized (non-crossover); double-blinded	a) Vitamin C-restricted diet + placebo (2 weeks); ozone (0.4 ppm)	2 hr	Exercise	Healthy on a low ascorbate diet (31)	18-35	Non-smokers.	Ozone-induced decrements in FEV_1 _and FVC were attenuated (p = 0.046 and p = 0.055 respectively), by vitamin C and vitamin E.
		b) Vitamin C-restricted diet + 250 mg of vitamin C, 50 IU of alpha-tocopherol, and 12 oz. of vegetable cocktail (2 weeks); ozone (0.4 ppm)						
		c) Vitamin C-restricted diet (1 week); air						
**Trenga, 2001, USA**	Randomized (crossover); double-blinded	a) Placebo; ozone (0.12 ppm)	45 min	Intermittent exercise	Adults with asthma (17)	18-39	Significant decrease (at least 8%) in FVC_1 _from baseline with SO_2 _challenge, non-smokers.	SO_2_-induced decrements in FEV_1 _and peak expiratory flow (PEF), and FEF_25-75 _were attenuated by vitamin C and vitamin E (p < 0.05).
		b) 400 IU vitamin E and 500 mg Vitamin C daily (4 weeks); ozone (0.12 ppm)						
		c) Placebo; air						
		d) 400 IU Vitamin E and 500 mg Vitamin C daily (4 weeks); air						
**Mudway, 2006, Sweden**	Randomized (crossover); double-blinded	a) Placebo; ozone (0.2 ppm)	2 hr	Intermittent exercise	Healthy ozone-sensitive (14)	21-27	Normal lung function, negative skin prick test, non-smokers, no history of allergy and/or lung disease.	No significant effect of antioxidants on ozone-attributable decrease in FEV_1 _(p > 0.05).
		b) 500 mg Vitamin C with 100 mg Vitamin E (7 days); ozone (0.2 ppm)						
		c) air						

* For crossover studies, each individual subjected to each condition; in non-crossover, the conditions were distributed between subjects.

**Table 2:** Studies assessing effect of antioxidant supplementation on lung function associated with *ambient* air pollutant exposures in intact humans

First author, Year, Country	Study design	Conditions*	Pollutant exposure duration	Anti-oxidant (dose) and duration	Population (N)	Age range	Inclusion/Exclusion criteria	Outcome
**Bucca, 1992, Italy**	Randomized (crossover); double-blinded	a) Placebo; 24-Hour average [pollutant]: SO_2 _142-159 (mcg/mol), total particulate 322-453 (mcg/mol), CO_2 _1.5-4.8 (ppm), NO_2 _139-178 (mcg/mol)	2 hours in 'acute' protocol; 4 days in 'chronic' protocol	Vitamin C (2 gm) once in 'acute' protocol and vitamin C (2 gm/day) for 4 days in 'chronic' protocol	Policemen (20)	24-37	Normal lung function tests, no history of asthma, respiratory tract infection in the six weeks preceding the study.	Pollutant-induced decrement in PC_25_MEF_50 _(airway reactivity) attenuated by vitamin C. in 'acute' protocol; Pollutant-induced decrement in PC_25_MEF_50 _attenuated by vitamin C in 'chronic' protocol (p < 0.001). Peak expiratory flow decrements also significantly attenuated by vitamin C (but p-value not reported).
		b)Anti-oxidant; 24-Hour average [pollutant]: SO_2 _142-159 (mcg/mol), Total particulate 322-453 (mcg/mol), CO_2 _1.5-4.8 (ppm), NO_2 _139-178 (mcg/mol)						
**Grievnik, 1998, The Netherlands**	Randomized	a) Placebo; 8-Hour average [ozone]: 101 (μg/m^3^)	70 days	Vitamin E (75 mg/day), Vitamin C (650 mg/day) and β-carotene (15 mg/day) for 3 months	Amateur cyclists (26)	16-41	Subjects with a range in exposure to ozone of less than 50 (μg/m^3^) were excluded for analysis.	Ozone-associated decrements in FVC, FEV_1_, and peak expiratory flow (PEF), were attenuated by vitamin E, vitamin C and β-carotene. No p-value provided.
		b)Anti-oxidant; 8-Hour average [ozone]: 101 (μg/m^3^)						
**Romieu, 1998, Mexico**	Randomized (crossover); double-blinded	a) Placebo; hourly average [pollutant]: ozone 67.3 (ppb), NO_2 _52.3 (ppb), PM_10 _76.5 (μg/m^3^) with placebo	6 months	Vitamin E (75 mg/day), Vitamin C (650 mg/day) and β-carotene (15 mg/day) for 130 days over 2 phases	Male street workers (47)	18-58	Working outdoors in the central area of Mexico City, No more than 5 cigarettes/day.	Ozone-associated decrease in FEV_1_, FVC and FEF_25-75 _attenuated by Vitamin C, Vitamin E and βeta-carotene. (first phase of study p values for all 3 parameters: p < 0.01; second phase of study p values for FVC and FEF_25-75 _p < 0.01 and p < 0.05 for FEV_1_).
		b)Anti-oxidant; hourly average [pollutant]: ozone 67.3 (ppb), NO_2 _52.3 (ppb), PM_10 _76.5(μg/m^3^)						
**Grievnik, 1999, The Netherlands**	Randomized (non-cross over); double-blinded	a)Placebo; 8-Hour average [ozone]: 84-88 (μg/m^3^)	15 weeks	Vitamin E (100 mg/daily), Vitamin C (500 mg/daily) for 15 weeks	Amateur and recreational cyclists (38)	17-58	Non smokers.	Ozone-associated decrease in FEV_1_, FVC attenuated by Vitamin C, and Vitamin E (p < 0.05).
		b)Anti-oxidant; 8-Hour average [ozone]: 84-88 (μg/m^3^)						
**Romieu, 2002, Mexico**	Randomized; double-blinded	a) Placebo; 24-Hour average [pollutant]: SO_2 _33 (ppb), PM_10 _56.68 (μg/m^3^), NO_2 _30 (μg/m^3^) ozone 32 (ppb)	12 weeks	Vitamin E (50 mg/day), Vitamin C (250 mg/day) for 12 weeks	Asthmatic children (158)	6-17	Not specified.	Ozone-associated decrements in FEF_25-75 _and PEF were attenuated by vitamin E and vitamin C (p < 0.05).
		b)Anti-oxidant; 24-Hour average [pollutant]: SO_2 _33 (ppb), PM_10 _56.68 (μg/m^3^), NO_2 _30 (μg/m^3^) ozone 32 (ppb)						

* For crossover studies, each individual subjected to each condition; in non-crossover, the conditions were distributed between subjects.

## Results

As illustrated by Figure 1, the original systematic search of six databases, using appropriate key words, resulted in 6012 literature entries. After duplicate studies were removed, a total of 3308 abstracts remained for assessment. After scanning abstracts to eliminate those not conforming to inclusion criteria, 64 papers remained for a full text analysis. After further scrutiny for conformity to specified inclusion criteria by the two independent reviewers, a total of 13 studies remained. Common reasons for exclusion at this stage were lack of lung function measurements or investigation of endogenous as opposed to supplemental antioxidants. The majority of selected studies were from the United States (6 studies), the Netherlands (3 studies) and Mexico (2 studies) with an additional study from Sweden and one from Italy. Vitamin C and ozone were the most common antioxidant and pollutant among the selected studies, respectively. Exposure length ranged from 45 minutes to 2 hours in the controlled conditions and from 2 hours to 6 months in the ambient pollution conditions. Tables 1 and 2 contain key information about the 13 studies that fully met inclusion criteria, including study design, study population, country and year of investigation, individual study inclusion and exclusion criteria, and relevant outcomes.

Table 1 summarizes eight studies that investigated the effects of antioxidant supplementation on lung function measurements, under controlled pollution conditions. Studies providing support for antioxidants, as well as those lacking such support, are noted and we did not find any evidence for publication bias. Five of the reported studies concluded that pollutant induced airway hyper-responsiveness and diminution in lung function which were attenuated by antioxidant supplementation. Mohsenin et al. [27] exposed eleven healthy subjects to one hour of controlled NO_2 _levels after vitamin C supplementation and reported a 21% decrease in the dose of Metacholine required to cause a 40% reduction (p < 0.04) in specific airway conductance (SGaw) after NO_2 _exposure without supplementation. This study further concluded that airway hypersensitivity induced by NO_2 _was almost entirely attenuated by pre-treatment with ascorbic acid (p < 0.04), relative to placebo. Peters et al. [28], in a similar study, reached the same conclusion after subject supplementation with Apocynin inhalations; specifically, this study concluded that Apocynin supplementation reduced the maximal percent fall from baseline (ΔMFEV_1_: fall in MFEV_1 _in response to two hours of ozone exposure) by 8.05%, compared to placebo (p = 0.025). Samet et al. [4] and Chatham et al. [29] exposed subjects to ozone under controlled conditions and after vitamin E and vitamin C supplementations, and both studies concluded that ozone-induced decrements in FVC were attenuated by these antioxidants. After 2 hours of ozone exposure, Samet et al. [4] observed that the O_3_-induced decreases in normalized FVC and FEV_1 _values in the supplemented group were 24% (p = 0.046) and 30% (p = 0.055) smaller, respectively, as compared with the placebo group; no significant effect on FEV_1_, FEF_25-75_, and SGaw was noted by Chatham (p > 0.05) after 2 hours of ozone exposure, but the effects of antioxidants on FVC was significant (p < 0.05). Finally, Trenga et al. [5] subjected seventeen asthmatic adults to 45 minutes of controlled ozone and air exposures followed by Sulfur dioxide challenge, after vitamin C and vitamin E supplementation. All pulmonary function tests measured were decreased significantly (p < 0.001) regardless of exposure atmosphere (air or ozone). However, subjects that were supplemented by Vitamin C & E responded less severely to post ozone exposure sulphur dioxide challenge than subjects given a placebo (FEV_1_: -1.2% vs. -4.4%, respectively; PEF: +2.2% vs. -3.0%, respectively; FVC: -2.15% vs. -2.51%, respectively, and FEF _25-75_: +2.0% vs. -4.3%, respectively), concluding that post ozone SO_2_-induced decrements in lung function measurements were attenuated by vitamin C and E supplementation.

Three studies failed to demonstrate such a moderating effect of antioxidants. Mudway et al. [3] observed significant (p < 0.01) decrements in FEV_1 _after 2 hours of ozone exposure in all fourteen subjects; however there were no significant effects of antioxidant supplementation (vitamin C and vitamin E) on ozone-attributable decrease in FEV_1 _(-8.5% with supplementation versus -7.3% with placebo; p > 0.05). Both independent trials of Hackney et al. [26] reached a similar conclusion after subject supplementation with varying vitamin E doses. The first trial reported a smaller decrease in lung function measurements (FVC, FEV_1_) in the supplemented group as compared to the placebo group, after 2 hours of controlled ozone exposure. More specifically, the reported post exposure ΔFVC with placebo was -0.22 ± 0.25 L as compared with -0.19 ± 0.40 L in the supplemented group without a statistical significance (t = 0.25, p > 0.05) and the reported post exposure ΔFEV_1 _with placebo was -0.27 ± 0.27 L as compared with -0.19 ± 0.35 L in the supplemented group without a statistical significance (t = 0.83, p > 0.05). During this trial, authors noted that the few vitamin supplemented male subjects demonstrated a potential beneficial effect and hence the second trial included only male subjects with similar exposure conditions as the first. However, this trial too failed to report significant effects of antioxidant supplementation on ozone-attributable decrease in FEV_1 _(p = 0.626), FVC (p = 0.661), FEF_50% _(p = 0.369), FEF_25% _(p = 0.225), and Total Lung Capacity (TLC, p = 0.221).

Table 2 summarizes the remaining 5 studies under ambient pollutant conditions. These studies each found that antioxidant supplementations attenuate the negative effects of ambient urban pollution on the lungs and airways. Romieu et al. [30] supplemented forty-seven male, Mexico city street workers with vitamin C, vitamin E and β-carotene for six months, while recording hourly average pollutant concentrations in an urban area in a two phase study. For the first phase, authors concluded that pollutant-associated decrease in FEV_1_, FVC and FEF_25-75 _are attenuated by supplementation with those antioxidants (p < 0.01 for all three parameters). Similar results were also reported in the second phase of the study (p < 0.01 for FVC and FEF_25-75 _and p < 0.05 for FEV_1_). A comparable study was conducted by Romieu et al. in 2002 [31] with 158 asthmatic children, followed-up for 12 weeks, as the study population. In this study, Romieu et al. observed significant (p < 0.05) differences in lung function decrements between supplemented and control groups for FEF_25-75 _and PEF, concluding that ozone-associated decrements in FEF_25-75 _and PEF were attenuated by vitamin E and vitamin C. Bucca et al. [32] conducted a two part study investigating both acute and long term effects of antioxidant supplementation in twenty policemen. The duration of exposure to the heavy car traffic city centre in the 'acute' protocol was 2 hours and this duration was 4 days in the 'chronic' protocol. In the first phase, authors reported that pollutant-induced decrement in Maximal mid-Expiratory Flow (MEF_50_) is attenuated by vitamin C supplementation. FVC and FEV_1 _remained nearly unchanged after NO_2 _exposure. In the second phase, authors reported that during placebo treatment the mean values of PEF were slightly but significantly decreased after exposure and were not significantly affected by exposure during vitamin C treatment. No quantitative statistical significances were provided for these results. Grievink et al. [33] reported that ozone-associated decrements in FVC, FEV_1_, and PEF are attenuated by vitamin C and vitamin E supplementation in twenty six amateur cyclists. Grievink and colleagues observed no significant effect on FVC, FEV_1_, PEF and MMEF (maximal mid-expiratory flow), due to an eight hour mean ozone exposure in the supplemented group; however all these parameters (except for MMEF) were decremented as a result of the exposure in the control group. Further, they reported the protective effects of supplementation (with the 95% confidence intervals) for each parameter as follows: FVC 2.08 (1.31 to 2.85) ml per μg/m^3 ^of ozone, FEV_1 _1.66 (0.62 to 2.70) ml per μg/m^3 ^of ozone, PEF 6.83 (3.17 to 10.49) ml per μg/m^3 ^of ozone. Finally, in a randomized and double blinded study, Grievink, et al. [34] measured lung functions of 38 "amateur and recreational" bicyclists before and after each training session for a period of 15 weeks. They concluded that Vitamin C and E supplementation provided a significant (p < 0.05) partial protection against ambient ozone induced decreases in FEV_1 _(median Δ: -0.95 ml per μg/m^3 ^of ozone for placebo and -0.01 ml per μg/m^3 ^of ozone for supplemented group) and FVC (median Δ: -1.25 ml per μg/m^3 ^of ozone for placebo and -0.42 ml per μg/m^3 ^of ozone for supplemented group) as compared to the placebo group.

## Discussion

We specifically aimed to limit our focus, as outlined above, based on the perceived gap between studies on intact humans with controlled anti-oxidant supplementation and the broader literature which is informative but is limited based on difficulties comparing human and animal physiology and/or by difficulties comparing observational and experimental studies. Our approach was strictly systematic; a complete and relevant list of search terms was utilized in multiple appropriate databases and an independent application of inclusion criteria by two authors collectively ensured the comprehensiveness of this systematic search. Our systematic approach distinguishes our work, particularly from the excellent effort of others. Romieu et al. [35] published an informative systematic review on air pollution, oxidative stress and dietary supplementation, with certain overlap with this systematic review; Florida-James et al. [36] discussed the potential negative impacts of various ambient pollutant exposures on the performance of athletes competing in Athens 2004. The current review however represents an update of previous reviews and is a formal and explicit search which systematically examined the literature and focused entirely on the potential role of anti-oxidant supplementations as opposed to also discussing biological and epidemiological evidence for the oxidative stress inducing role of air pollutants. Accordingly, we expect that our approach is most helpful to those considering practical decisions and possibly guidelines, as well as researchers considering the need for further related work, regarding anti-oxidant supplementation to attenuate air pollution effects.

An immediate consistency in results is not apparent within studies conducted under controlled conditions (Table 1). Even though 5 out of 8 studies in suggested a potential benefit for antioxidant supplementation, such results are far from unanimous and are contradicted by interesting studies that did not make our inclusion criteria. For example, as stated above, Mudway and colleagues [20] subjected 30 volunteers (15 health and 15 mild asthmatics) to 2 hours of controlled ozone exposures; they observed that the asthmatic group had significantly (p < 0.01) lower basal Ascorabte concentrations extracted from their proximal and distal respiratory tract lining fluid as compared with the control group. However, this depletion in basal antioxidant concentrations was not associated with a significantly higher ozone induced neutrophilia or decrement in FEV_1 _as compared with the control group. This suggests a potential discrepancy between the roles of supplemented antioxidants compared to basal concentrations and warrants future studies to differentiate distinguished benefits from each.

Despite the small number of studies conducted to date that fall within our inclusion criteria, the general consistency of studies in Table 2, compromising of research conducted in ambient pollution conditions is noteworthy. Arguably, these studies provide the most directly interpretable results, in terms of implementation of available data in public health recommendations; all such studies reported uncontrolled (ambient) pollutant conditions that were directly representative of daily life in these locations. Such realistic exposures are more representative and informative than studies conducted under controlled exposure conditions, such as those in Table 1. All of the uncontrolled exposure studies concluded that a potential benefit for antioxidant supplementation in attenuating the negative effects of air pollution is evident; these findings are consistent with previous studies investigating the role of various anti-inflammatory interventions, for example azithromycin [37], indomethacin [38], cyclooxygenase metabolites [39] and budesonide [40] on pollution induced lung outcome. However, such consistency does not refute the need for more research on the subject. For example, of all the studies in Table 2, only one study [32] analysed the role of antioxidant supplementation on attenuating the negative health effects of combined exposure to measured pollutants. In other words, all other studies only focused on the ability of supplements to lessen the effects of only one of the measured pollutants (such as ozone), even though in reality humans are continuously exposed to combined levels of all relevant pollutants and research with public health applicability necessities an investigation of the combined effects of these pollutants and the potential role of supplementation. Furthermore, the lack of personal exposure data in most studies is problematic in that exposure misclassification may occur under such circumstances. Finally, Grievink [33] was the only study that focused particularly on the effects of outdoor particulate matter (PM) exposure, despite significant morbidity and mortality associated with such exposures as outlined above [1].

## Conclusions

Of the 13 studies that were reviewed for the current study, vitamin C, Vitamin E and B-carotene were examined for their roles in attenuating the adverse effects of pollutant. There were conflicting results based on the varying controlled and uncontrolled exposures, yet there was a trend for potential benefit for antioxidant supplementation in ten of the 13 studies. Meanwhile the effects on morbidity and mortality are still unclear. Therefore future research in humans will need to focus on selecting appropriate exposure materials and length of time to quantify the benefits of antioxidant supplementation.

## Competing interests

The authors declare that they have no competing interests.

## Authors' contributions

AYT performed latter stages of analysis and wrote manuscript. KC performed earlier stages of analysis and edited manuscript. CC conceptualized the project, reviewed abstracts as described in the manuscript, supervised the project overall, and edited manuscript. All authors read and approved the final manuscript.

## Pre-publication history

The pre-publication history for this paper can be accessed here:

http://www.biomedcentral.com/1471-2458/11/532/prepub

## Supplementary Material

Additional file 1 **Search Strategy**. This additional file is a *.doc file format. It contains all the details for the search strategy performed for the research article. The document is 8 pages. Click here for file
