# Supplementary material for: Modification by antioxidant supplementation of changes in human lung function associated with air pollutant exposure: A systematic review
Source: BMC Public Health. 2011 Jul 5;11:532. doi: 10.1186/1471-2458-11-532 (PMC3158771; doi:10.1186/1471-2458-11-532)
Supplement: Additional file 1 — Search Strategy. This additional file is a *.doc file format. It contains all the details for the search strategy performed for the research article. The document is 8 pages. [file 1471-2458-11-532-S1.DOC]

**Appendix I.** Search strategy for EMBASE (1980 to 2009 week 16) and Ovid MEDLINE(R) 1950 to April Week 4 2009

1. exp respiratory system/
2. exp respiratory tract diseases/
3. ((respiratory or trache$ or lung or airway$ or pulmon$ or bronch$ or sinus$ or pharyn$) adj5 diseas$).mp.
4. exp respiratory function tests/
5. ((respiratory or pulmon$) adj5 function).mp.
6. plethysmography.mp.
7. spirometry.mp.
8. or/1-7
9. exp Air Pollution/
10. exp Air Pollutants/
11. PARTICULATE MATTER.mp.
12. (air adj10 pollutant$).mp.
13. (air adj10 pollution$).mp.
14. nitrogen dioxide.mp.
15. ozone.mp.
16. (hydrogen sulfide or hydrogen sulphide).mp.
17. exp inhalation exposure/ or exp occupational exposure/
18. (diesel or gasoline or petro$ or sulfur dioxide).mp.
19. or/9-18
20. exp Antioxidants/
21. (free adj5 radical$).mp.
22. acetylcystein$.mp.
23. tocopherol$.mp.
24. Melatonin.mp.
25. (caroten$ or retinol).mp. [mp=title, original title, abstract, name of substance word, subject heading word]
26. Ubiquinone/
27. (Superoxide adj3 Dismutase).mp.
28. peroxidase$.mp.
29. ubiquin$.mp.
30. (coenzyme adj3 q).mp.
31. vitamin$.mp.
32. ascorb$.mp.
33. antioxidant$.mp.
34. or/20-33
35. 8 and 19 and 34
36. animals/
37. humans/
38. 36 not (36 and 37)
39. 35 not 38

**Appendix II.** Search strategy for BIOSIS Previews 1969 to 2009

1. ((respiratory or pulmon$) adj5 function).mp.
2. plethysmography.mp.
3. spirometry.mp.
4. exp "Respiratory system - Physiology and biochemistry"/ or exp Respiratory System Disease/ or exp Respiratory System/ or exp Respiratory system - Pathology/ or exp Pulmonary Medicine/ or exp Respiration/
5. ((respiratory or trache$ or lung or airway$ or pulmon$ or bronch$ or sinus$ or pharyn$ or pleur$) adj5 (diseas$ or function$)).mp.
6. (lung adj3 (compliance or volume$)).mp.
7. asthma.mp.
8. ((lung or respirat$ or bronch$) adj3 (hyperreact$ or hypersens$)).mp.
9. ((interstitial or obstructive) adj3 lung).mp.
10. emphysema.mp.
11. (pulm$ adj3 (eosinophilia or fibrosis or neoplasm$)).mp.
12. or/1-11
13. PARTICULATE MATTER.mp.
14. (air adj10 pollutant$).mp.
15. (air adj10 pollution$).mp.
16. nitrogen dioxide.mp.
17. ozone.mp.
18. (hydrogen sulfide or hydrogen sulphide).mp.
19. exp "Pollution Assessment Control and Management"/ or exp "Public health - Air, water and soil pollution"/
20. ((inhal$ or occupation$) adj3 exposure$).mp.
21. ((vehicle$ or auto$ or car$) adj3 emission$).mp.
22. diesel.mp.
23. gasoline.mp.
24. petro$.mp.
25. (sulf$ adj3 (dioxide or anhydride)).mp.
26. or/13-25
27. acetylcystein$.mp.
28. tocopherol$.mp.
29. Melatonin.mp.
30. (caroten$ or retinol).mp.
31. peroxidase$.mp.
32. ubiquin$.mp.
33. (coenzyme adj3 q).mp.
34. vitamin$.mp.
35. ascorb$.mp.
36. antioxidant$.mp.
37. bilirubin$.mp.
38. (Butylated adj3 (Hydroxyanisole or Hydroxytoluene)).mp.
39. Canthaxanthin$.mp.
40. Catalase$.mp.
41. (free adj3 radical$).mp.
42. Quercetin.mp.
43. flavon$.mp.
44. Selenium.mp.
45. uric acid.mp.
46. Superoxide Dismutase.mp.
47. or/27-46
48. 12 and 26 and 47

**Appendix III.** Search strategy for Web of Science (1965 to 2009 week 16).

1. TS=((lung* or bronch* or respirat* or airway* or trach* or laryngeal* or pleural* or pulmon* or mediastin*) SAME (neoplasm* or cancer* or malignanc*))
2. TS=((lung* or bronch* or respirat* or airway* or trach* or laryngeal* or pleural* or pulmon* or mediastin*) SAME (disease*))
3. TS=((lung* or bronch* or respirat* or airway* or trach* or laryngeal* or pleural* or pulmon* or mediastin*) SAME (hypersens* or reactivit*))
4. TS=respiratory system*
5. TS=((respiratory or lung or pulmonary) SAME (function* or volume* or compliance*))
6. TS=(plethysmography or spirometry)
7. TS=(pulmonary fibrosis) or TS=(pulmonary eosinophilia) or TS=(interstitial lung disease*) or TS=emphysem* or TS=(obstructive lung disease*) or TS=asthma
8. #7 OR #6 OR #5 OR #4 OR #3 OR #2 OR #1
9. TS=Superoxide Dismutase or TS=uric acid* or TS=Selenium or TS=flavon* or TS=Quercetin or TS=free radical* or TS=Catalase* or TS=Canthaxanthin* or TS=(butylated hydroxyanisole or butylated hydroxytoluene) or TS=bilirubin* or TS=ascorb* or TS=vitamin* or TS=coenzyme q or TS=ubiquin* or TS=peroxidase* or TS=(caroten* or retinol) or TS=melatonin* or TS=tocopherol* or TS=acetylcystein*
10. TS=antioxidant*
11. #10 OR #9
12. TS=(sulf* SAME (dioxide or anhydride)) or TS=petro* or TS=gasoline or TS=(diesel) or TS=((vehicle* or auto* or car*) SAME emission*) or TS=((inhal* or occupation*) SAME exposure*)
13. TS=((hydrogen sulfide) or (hydrogen sulphide)) or TS=(ozone) or TS=(nitrogen dioxide) or TS=(particulate matter*) or TS=(air pollutant*)
14. #13 OR #12
15. #11 AND #8
16. #15 AND #14

**Appendix IV.** Search strategy for TOXNET (1965 to 2009 Week 16)

1. ( ( ( pulmonary OR lung ) ) AND ( ( function OR physiology ) OR volume ) )
2. ( disease* AND ( ( lung ) ) )
3. ( spirometry )
4. ( ( asthma ) OR ( emphysema ) OR ( obstructive ( lung ) ) )
5. ( ( asthma ) OR ( emphysema ) OR ( obstructive ( lung ) ) OR ( ( pulmonary fibrosis ) ) )
6. ( ( pulmonary OR lung ) AND ( ( neoplasm OR neoplasms ) OR ( cancer OR neoplasms ) ) )
7. ( ( pulmonary OR lung ) AND neoplasma )
8. ( ( pulmonary OR lung ) AND ( neoplasm OR neoplasms ) )
9. ( #6 OR #5 OR #3 OR #2 OR #1 )
10. ( " air pollution " )
11. pollut* AND ( air )
12. ( ( gasoline OR 8006-61-9 [rn] ) OR diesel OR petro* )
13. ( #11 OR #12 )
14. ( ( ( vehicle OR vehicles ) OR ( car OR automobiles ) ) AND emission )
15. ( #13 OR #14 )
16. ( #9 AND #15 )
17. ( antioxidant OR antioxidants )
18. ( acetylcysteine OR mucomyst OR "mercapturic acid" OR acetadote OR respaire OR parvolex OR fluimucil OR fluimucetin OR broncholysin OR airbron OR 616-91-1 [rn] )
19. ( tocopherol OR tocopherols OR methyltocols OR 1406-66-2 [rn] )
20. ( melatonin OR melatonine OR 73-31-4 [rn] )
21. ( carotene OR carotenoids OR phytoxanthins OR 36-88-4 [rn] )
22. ( peroxidase OR lactoperoxidase OR myeloperoxidase OR "horseradish peroxidase" OR 9003-99-0 [rn] )
23. ( ubiquinone OR ubiquinones OR "coenzyme q" OR 1339-63-5 [rn] )
24. ( #17 OR #18 OR #19 OR #20 OR #21 OR #22 OR #23 )
25. ( #24 AND #16 )

**Appendix V.** Search Strategy for Environmental Sciences & Pollution Management (1967 to 2009 week 16),

(((air pollut*) or ozone or (particulate matter)) or ((occupation* exposure*) or (inhal* exposure*) or (auto* emission)) or ((vehicle* emission*) or (car* emission*))) and((((respiratory disease*) or (lung disease*) or (airway disease*)) or (pulm* disease*)) or(((resp* function*) or (pulm* function*) or (lung function*)) or (lung volume*))) and((antioxidant*) or((acetylcystein* or tocopherol* or Melatonin) or (caroten* or retinol or peroxidase*) or (ubiquin* or vitamin*)))
